# Post-traumatic cluster headache: a clinical phenotype study of 16 patients

**DOI:** 10.1186/1129-2377-14-S1-P46

**Published:** 2013-02-21

**Authors:** G Lambru, CK Chan, MS Matharu

**Affiliations:** 1 Headache Group, UK

## Introduction

Cluster headache (CH) due to head trauma seems to be an extremely rare entity. To date only two cases of new onset CH that fulfill the criteria for post-traumatic headache have been described.

## Aims

Describe the phenotype and response to treatments of a series of post-traumatic CH patients.

## Methods

Sixteen cases fulfilling the International Headache Society (IHS) criteria for post-traumatic headache with the CH phenotype were identified out of a cohort of 302 CH patients (chronic: 64%) seen between 2007 and 2011. Details on the head injuries, along with clinical information on CH were collected.

## Results

Five percent of our sample of patients had CH secondary to head trauma. All patients developed a chronic form of post-traumatic headache. Fourteen patients had chronic CH and 2 episodic CH (M:F=2:1). The median age of onset of CH was 31 years (range: 10-54). Eight patients (50%) reported a correspondence between the trauma site and the CH side. The most frequent circumstances of head traumas included: brawls in 5 patients (31%) and sport accidents in 4 (25%). No atypical clinical features were noticed. Remarkably, 3 patients (19%) had familial CH. Sumatriptan 6 mg injection was effective in 15 patient; high dose and flow rate oxygen was effective in 5 patients. Verapamil was effective in 7 patients.

## Conclusion

This is the largest series of post-traumatic CH. This condition does not differ from the idiopathic form in terms of phenotype and response to treatment. The frequent occurrence of head injuries during brawls, may suggest a risk-taking trait in some CH patients. The high proportion of familial CH in this series might suggest that exogenous factors, such as a head injury, may alter the homeostasis of the trigeminovascular system giving rise to CH particularly in genetically susceptible individuals.

